# Association of PM2.5 exposure with 10-year atherosclerotic cardiovascular disease risk: a mediation analysis of blood and urinary biomarkers

**DOI:** 10.3389/fpubh.2026.1799605

**Published:** 2026-04-10

**Authors:** Ying Han, Mengdan Liang, Tingting Xu, Min Pan, Jin Gong, Bangbang Huang, Liangdi Xie, Xiaoxu Xie, Huashan Hong

**Affiliations:** 1 Department of Geriatrics, Fujian Institute of Geriatrics, Fujian Key Laboratory of Vascular Aging (Fujian Medical University), Fujian Clinical Research, China Center for Senile Vascular Aging and Brain Aging, Fujian Medical University Union Hospital, Fuzhou, China; 2 Department of Epidemiology and Health Statistics, School of Public Health, Fujian Medical University, Fuzhou, China; 3 Medical Department, Jinjiang Municipal Hospital, Shanghai Sixth People's Hospital Fujian, Quanzhou, China; 4 Department of Geriatrics, The First Affiliated Hospital of Fujian Medical University, Fuzhou, China; 5 Fujian Hypertension Research Institute, The First Affiliated Hospital of Fujian Medical University, Fuzhou, China; 6 Fujian Provincial Sub-Center of National Clinical Research Center for Geriatric Disorders, The First Affiliated Hospital of Fujian Medical University, Fuzhou, China

**Keywords:** air pollution, atherosclerotic cardiovascular disease, China-PAR, fine particulate matter, mediation analysis

## Abstract

**Aims:**

To explore the relationship of fine particulate matter (PM_2.5_) exposure with 10-year risk of atherosclerotic cardiovascular disease (ASCVD), explore effect modifiers, identify susceptible populations, and examine potential mediating factors.

**Methods and results:**

A cross-sectional study among 9,539 residents of Fujian Province, China, from May 2021 to January 2022 was administered. PM_2.5_ exposure was estimated using the China High Air Pollutants (CHAP) dataset with a spatial resolution of 1 km from May 2021 to January 2022. ASCVD risk was predicted utilizing the China-PAR model. We performed weighted linear mixed-effects models to assess the association of exposure to PM_2.5_ with 10-year risk of ASCVD. Potential effect modifiers were assessed using stratified analyses and interaction analysis. We used mediation analyses to explore the role of blood and urine indicators in this association. An elevated risk of 10-year ASCVD was found in association with PM_2.5_ exposure. For every 5 μg/m^3^ rise in PM_2.5_, 10-year ASCVD risk increased by 3.246 (95% CI: 2.602, 3.889). Age, smoking, alcohol consumption, and hypertension modified the association of exposure to PM_2.5_ with the risk of ASCVD. Glycosylated hemoglobin, fasting blood glucose, and blood uric acid partially mediated this association.

**Conclusion:**

Exposure to PM_2.5_ is correlated with the elevated 10-year ASCVD risk, with blood glucose, and uric acid potentially acting as mediators.

## Graphical abstract

**Figure:**
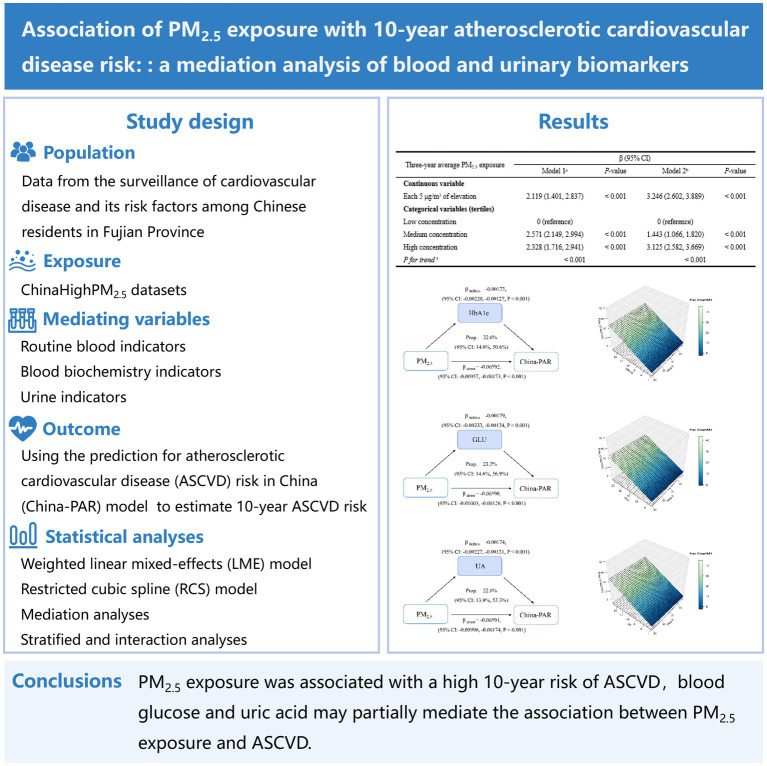

## 1 Introduction

Cardiovascular diseases (CVDs) rank as the leading cause of death and disability worldwide, imposing a substantial health and economic impact (1). At present, the morbidity and mortality of CVDs in China show an increasing trend, which is closely related to the changes in the lifestyle, dietary structure of Chinese residents and environmental changes (2). In particular, mortality due to atherosclerotic cardiovascular disease (ASCVD), primarily ischemic heart disease and ischemic stroke, has risen notably (3). Between 1990 and 2016, the share of ASCVD in overall cardiovascular deaths increased by 21%, and the average number of deaths per year grew by 1.4 million (2), the prevention and control situation has becoming increasingly critical.

Air pollution stands out as a crucial environmental hazard affecting global health, and particulate matter pollution is the top contributor to the global burden of disease in 2021 (4). The World Health Organization (WHO) reports that around 7 million global deaths annually are linked to air pollution exposure, with CVDs accounting for half of these, surpassing other modifiable CVD risk factors like smoking, hypertension, hyperlipidemia, and diabetes mellitus (5). A review of pollution and the heart suggests that air pollution is recognized as a significant factor in cardiovascular diseases and associated risk factors (6). A number of epidemiological investigations have uncovered that air pollution is correlated with elevated CVDs risk (7–9). However, previous studies have primarily focused on heavily polluted areas in northern China (such as Beijing), while evidence from moderately polluted coastal regions in the south remains relatively scarce.

The potential mechanisms linking air pollution to ASCVD risk have attracted research attention, however, they are not yet fully understood. Air pollution comprises a mixture of particulate matter and gases, with particulate matter containing black carbon, metals, and various organic compounds. Upon respiration of these particles, macrophages and epithelial cells are stimulated to release pro-inflammatory cytokines, which initiates an inflammatory reaction in the body, affecting the function of numerous body systems (10, 11). Previous animal studies have shown that particulate matter components promote atherosclerotic plaque growth and increase plaque rupture vulnerability (12, 13). The current explanations about the health hazards of air pollution are mainly that air pollution exposure leads to oxidative stress (14), inflammatory response (15, 16), endothelial dysfunction (17), atherosclerotic thrombosis (18), mitochondrial dysfunction (19), metabolic abnormality (20), imbalance of the autonomic nervous system (21, 22), and so on. However, the definite mechanism about the effect of air pollution on ASCVD needs to be explored by further research, and mediational analysis seemed to has provided a new way of thinking to explore the mechanism. Mediation analysis, which splits the overall exposure effect into indirect and direct components, not only can uncover if exposure leads to the outcome but also clarify the process by which it happens (23). Mediation analysis has served as a crucial statistical method for understanding the potential mechanisms of exposure-outcome relationships. Previous studies have primarily focused on research-grade biomarkers (such as IL-6, TNF-α, etc.), which require specialized assays and high costs The mediating role of routinely accessible clinical biomarkers (such as complete blood count, blood biochemistry, and urinalysis) in the cardiovascular effects of air pollution remains incompletely understood.

Cardiovascular risk prediction model provides a convenient and reliable method for assessing cardiovascular health (24). The Framingham Heart Study (FHS) first developed a cardiovascular disease risk assessment model in 1976 (25). Many new models have been developed in recent decades (26). Current cardiovascular disease risk assessment models include Systematic Coronary Risk Estimation (SCORE) model (27), Prospective Cardiovascular Münster (PROCAM) score (28), QRISK score (QRISK) model (29), Reynolds Risk Score (30), among others. However, most of these models were developed based on foreign populations and are not suitable for the overall risk assessment of CVDs in China. In 2016, leveraging large-sample cohort data from the China ASCVD Risk Prediction (China-PAR) study, Chinese scholars established the China-PAR model to estimate the 10-year and lifetime risks of cardiovascular disease (31), and proposed risk stratification standards appropriate for the Chinese population. China-PAR is a prospective cohort study from China, incorporating 12 variables for an assessment model of the risk of ASCVD occurrence that combines geographic, urban and rural, and personal health indicators with prevalence characteristics of CVDs, and has been validated and shown to have strong internal and external consistency.

Therefore, based on the data collected from the surveillance of cardiovascular disease and its risk factors among Chinese residents in Fujian Province and utilizing the China-PAR score to estimate the risk of ASCVD, the study aimed to (1) investigate the association of PM_2.5_ with ASCVD in a moderate-pollution coastal region of southeastern China; (2) search for the potential effect modifiers, and (3) systematically evaluate the mediating roles of routine clinically accessible biomarkers in PM_2.5_ cardiovascular effects.

## 2 Methods

### 2.1 Population and study design

In current study, we conducted a cross-sectional research design. All participants came from cardiovascular disease and its risk factor surveillance in Chinese residents. Detailed information about cardiovascular disease and its risk factor surveillance in Chinese residents can be found in Supplementary material. Our study was conducted with data from May 2021 to January 2022 in Fujian province. A total of 9,790 subjects were surveyed during this period. We excluded participants diagnosed with CAD, as well as those without accurate data on questionnaire completion time and PM_2.5_ matching. Ultimately, the analysis comprised a total of 9,539 participants. The translated questionnaire can be found in Supplementary file. Each participant included in the study gave written informed consent before data collection.

### 2.2 Assessment of PM_2.5_ exposure levels

PM_2.5_ exposure data are obtained from the China High Air Pollutants (CHAP) dataset, which provides comprehensive, long-term, high-precision, high spatial and temporal resolution remote sensing dataset on near-surface for ground-level air pollutants across China. Our study used the PM_2.5_ dataset (China High PM_2.5_) from the CHAP datasets (32, 33). PM_2.5_ exposure values were extracted for each year based on the latitude and longitude coordinates of the study subjects using ArcMap 10.7 software using the nearest neighbor method. The estimation of PM_2.5_ exposure was based on the average exposure level of each participant in the 3 year prior to the baseline survey. Exposure was also estimated in the sensitivity analysis section using the average exposure to PM_2.5_ for each participant in 1 and 5 years before the baseline survey. More detailed information on exposure can be found in the attached document.

### 2.3 Assessment of outcome

The 10-year predicted risk of Atherosclerotic Cardiovascular Disease (ASCVD) was calculated by China-PAR project (China ASCVD Risk Prediction). Detailed information on the China-PAR risk assessment model and examples is provided in Supplementary material. According to Chinese guidelines, participants were classified as having a low (less than 5%), intermediate (5–9.9%) and high (greater than or equal to 10%) 10-year ASCVD risk, as determined by their ASCVD risk score (34).

### 2.4 Mediation variables

In our study, 14 mediating variables were considered, including routine blood, blood biochemistry, and urine indicators. Of these, routine blood indicators include white blood cells, red blood cells, hemoglobin and neutrophils, platelets. Blood biochemistry indicators include fasting blood glucose, glycosylated hemoglobin, triglycerides, low-density lipoprotein cholesterol (LDL-C), creatinine, uric acid (UA) and potassium. Urine indicators include urinary creatinine and urinary microalbumin. A description of the mediating variables can be found in Supplementary Table S4.

### 2.5 Covariates

Covariate selection was based on previous literature considering variables such as demographic characteristics of the study population, lifestyle (35–38). Including marriage (married/unmarried and other), education (not attending school, elementary school, junior high school, high school/secondary school, and college/bachelor’s degree and above), alcohol consumption (yes/no), and per capita disposable income of the household (under 10,000 RMB, 10,000–20,000 RMB, 20,000–30,000 RMB, 30,000–50,000 RMB, 50,000–100,000 RMB, and over 100,000 RMB).

### 2.6 Statistical analyses

As most continuous variables were not normally distributed, Kruskal-Wallis H tests were utilized for continuous variables [shown as median (interquartile spacing [IQR])]. Chi-square tests were conducted for categorical variables [shown as frequency (percentage)] to describe baseline characteristics.

The data were weighted according to a multi-stage unequal probability sampling design, and a weighted linear mixed-effects (LME) model was employed to estimate the relationship of exposure to PM_2.5_ with 10-year risk of ASCVD, with counties and districts as random effects. PM_2.5_ was first analyzed as a continuous variable, specifically, raw PM_2.5_ concentrations were divided by 5 prior to analysis to scale the regression coefficient *β* to directly represent the effect size per 5 μg/m^3^ increment. And then the tertiles of PM_2.5_ exposure were used as categorical variable, designating the group with the least exposure as the reference category. Trend tests were conducted for the likelihood of monotonically increasing or decreasing effect size, specifically, the tertile levels of PM_2.5_ exposure concentration were coded as 1, 2, and 3 sequentially, and the codes were included in the model as measures to be fitted, and then the hypotheses were tested as the PM_2.5_ exposure concentration variable for continuous data to obtain the *p* value of the trend test. To models were constructed. Model 1 represents unadjusted model. Model 2 incorporates adjustments for marriage, education, alcohol consumption, and per capita household disposable income. Exposure-response relationships of exposure to PM_2.5_ with 10-year ASCVD risk were estimated utilizing restricted cubic splines (RCS) model.

Stratification analyses were performed by age (<60 years/ ≥60 years), sex (male/female), residence (rural/urban), smoking (yes/no), alcohol consumption (yes/no), hypertension (yes/no), and diabetes mellitus (yes/no) to assess potential effect modification. And test the modification effect’s significance by incorporating product terms (PM_2.5_ exposure × effect modifier) into models.

We used the “mediation” package in R to perform mediation analyses to assess whether routine blood indicators, blood biochemical indicators, and urine indicators mediated the relationship of exposure to PM_2.5_ with 10-year ASCVD risk in order to preliminarily explore how PM_2.5_ might lead to cardiovascular damage. Specifically, we fitted three models: the first model estimated the exposure-outcome association with covariate adjustment, the second model assessed the mediator-outcome association with covariate adjustment, and the third model estimated the exposure-outcome association with adjustments for both mediator and covariates. The direct impact refers to how exposure influences the outcome without involving mediator. The indirect impact denotes the influence of exposure on the outcome via a mediator. The total impact is the combination of both direct and indirect impacts. The mediator percentage was calculated as


$$\text{Prop}.=\frac{\text{indirect effect}}{\text{Total effect}}\times 100\%$$

We employed 500-times bootstrap to obtain the 95% confidence intervals. 3D surface maps were used to estimate the exposure-response relationships concerning PM_2.5_, mediator and 10-year risk of ASCVD (“Plot 3D” package in R software).

Additionally, several sensitivity analyses were conducted to examine the robustness of our results: (1) the average PM_2.5_ exposure level in the 1 and 5 years before the baseline survey were used to assess the reliability of the main results; (2) based on model 2, additional adjustments were made for dietary score. We constructed a weighted dietary score based on the intake and frequency of six dietary factors: vegetables, fruits, meat, fish, eggs, and milk. First, using the China-PAR risk score as the dependent variable and the intake frequency of each of the six food categories as independent variables, we established multiple linear regression models to determine the strength of association (*β* coefficient) between each dietary factor and 10-year risk of ASCVD risk. Subsequently, individual dietary scores were calculated using a weighted summation approach according to the following formula: dietary score = βvegetable∗ vegetable + βfruit∗ fruit + βmeat∗meat + βfish∗ fish + βegg∗ egg+βmilk∗ milk; (3) based on model 2, additional adjustments were made for CO, NO_2_, O_3_, SO_2_, and PM_1_.

The criterion for statistical significance was set at *p* < 0.05, utilizing a two-sided test approach. Analyses were conducted by R (version 4.3.1).

## 3 Results

### 3.1 Descriptive results

Among the 9,539 participants, the median age [inter-quartile range] was 43 [30, 57] years, with 4,703 (49.3%) males and 4,836 (50.7%) females. Among all participants, 72.1% of the study subjects were married, 13.3% were hypertensive, 3.5% were diabetic, 22.8% were smokers, 31.3% were alcohol drinkers, 62.8% lived in rural areas, and 2.9% had a family history of CVDs. The median [inter-quartile range] 3-year PM_2.5_ exposure level of the study subjects was 23.3 [22.2, 25.5], and the median [inter-quartile range] China-PAR scores of the study subjects was 1.90 [0.32, 8.33] (Table 1). Supplementary Table S5 displays the baseline characteristics of the study population, which were categorized according to the China-PAR score of the Chinese guidelines.

**Table 1:** Descriptive statistic of the study participants and PM_2.5_.

Variable	*N* = 9,539
Age (years)^a^	43 [30, 57]
Gender, *n* (%)	
Male	4,703 (49.3)
Female	4,836 (50.7)
Marital status, *n* (%)	
Married	6,879 (72.1)
Unmarried/Others	2,660 (27.9)
Hypertension, *n* (%)	
No	8,269 (86.7)
Yes	1,270 (13.3)
Diabetes mellitus, *n* (%)	
No	9,203 (96.5)
Yes	336 (3.5)
Smoking status, *n* (%)	
No	7,358 (77.2)
Yes	2,173 (22.8)
Drinking status, *n* (%)	
No	6,547 (68.7)
Yes	2,985 (31.3)
Address, *n* (%)	
Rural	5,987 (62.8)
Urban	3,552 (37.2)
Family history of cardiovascular disease, *n* (%)	
No	9,259 (97.1)
Yes	272 (2.9)
Antihypertension drugs intake, *n* (%)	
No	8,389 (87.9)
Yes	1,150 (12.1)
Education, *n* (%)	
Illiteracy	1,190 (12.5)
Primary school	1,633 (17.1)
Junior high school	2,221 (23.3)
High school/trade school	1,766 (18.5)
Junior college or above	
Per capita disposable income, *n* (%)	
[0, 10,000)	1,140 (12.0)
[10,000, 20,000)	2,043 (21.4)
[20,000, 30,000)	2,182 (22.9)
[30,000, 50,000)	1,881 (19.7)
[50,000, 100,000)	1,496 (15.7)
[100,000, Inf)	797 (8.4)
Systolic blood pressure (mm Hg)^a^	127 [115, 141]
Total cholesterol (mmol/L)^a^	4.82 [4.22, 5.55]
High-density lipoprotein cholesterol (mmol/L)^a^	1.38 [1.19, 1.61]
Waist circumference (cm)^a^	79.7 [72.0, 87.0]
Body mass index (kg/m^2^)^a^	23.4 [21.1, 26.0]
One-year average PM_2.5_ exposure concentration (μg/m^3^)^a^	21.2 [19.9, 22.8]
Three-year average PM_2.5_ exposure concentration (μg/m^3^)^a^	23.3 [22.2, 25.5]
Five-year average PM_2.5_ exposure concentration (μg/m^3^)^a^	25.4 [24.0, 27.7]
China-PAR scores^a^	1.90 [0.32, 8.33]

Note: PM_2.5_, fine particulate matter; China-PAR, risk prediction of atherosclerotic cardiovascular disease in China.

a Data are expressed as median [Inter-quartile range].

### 3.2 Association of PM_2.5_ exposure with 10-year risk of ASCVD

**Figure 1:**
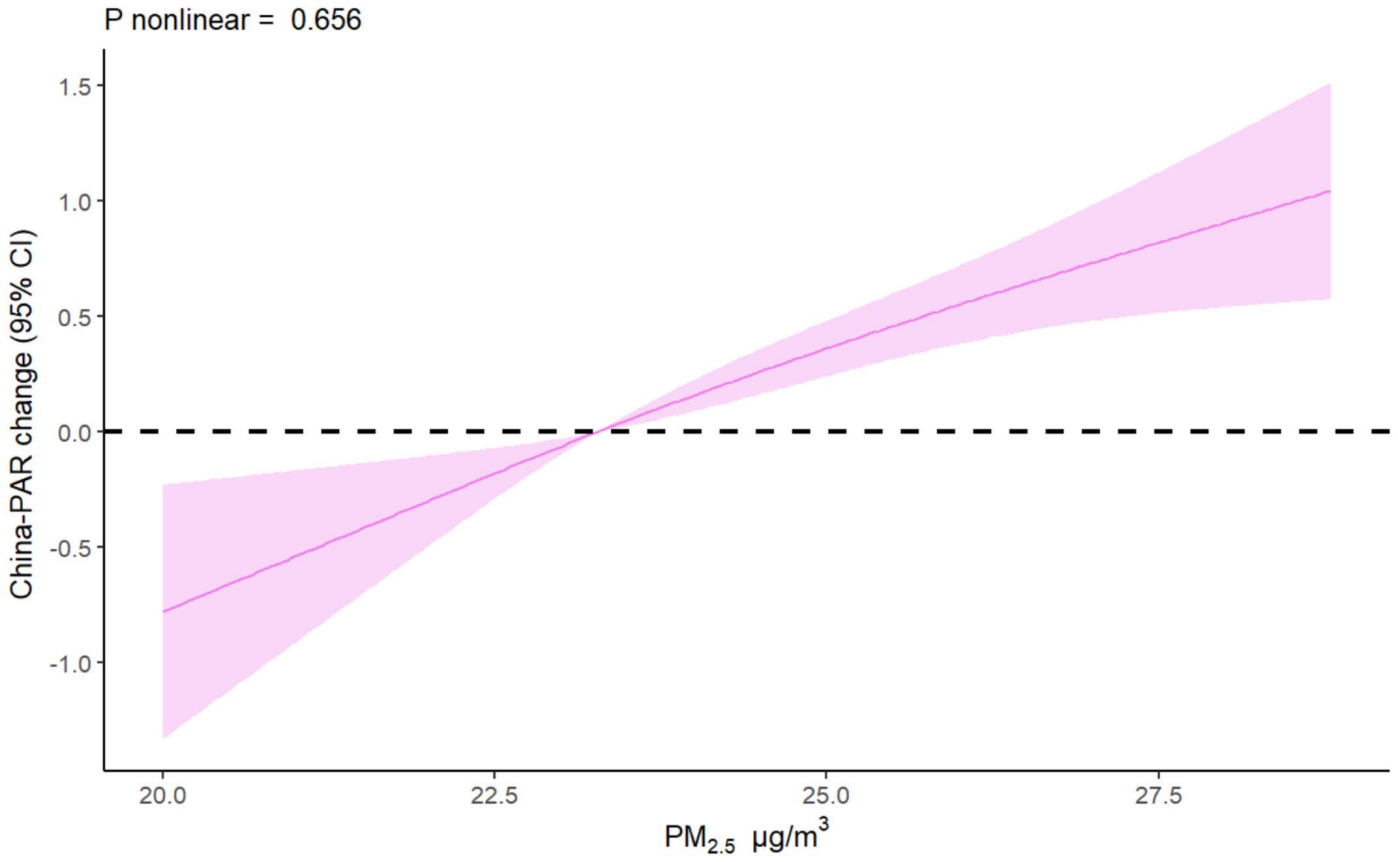
Exposure-response function analysis of PM_2.5_ exposure levels and China-PAR scores. Restricted cubic spline was used to estimate the exposure-response relationship (solid line) and its 95% confidence interval (shaded). Model was adjusted for marital status, education level, drinking status, and per capita disposable income. PM_2.5_, fine particulate matter; China-PAR, risk prediction of atherosclerotic cardiovascular disease in China; CI, confidence interval.

PM_2.5_ exposure may elevate 10-year ASCVD risk after adjusting for confounding factors. For every 5 μg/m^3^ increase in PM_2.5_ exposure, the China-PAR risk score increased by an average of 3.25 points, indicating an increased 10-year ASCVD risk (*β* = 3.25, 95% CI: 2.60, 3.89). Then we categorize PM_2.5_ exposure into three levels, compared with low tertiles of PM_2.5_ exposure individuals, participants in higher tertiles of PM_2.5_ exposure (moderate: *β* = 1.44, 95% CI: 1.07, 1.82; high: *β* = 3.13, 95% CI: 2.58, 3.67) has higher risk of 10-year ASCVD (*p* for trend less than 0.05) after adjusted for covariates (Table 2). RCS analysis reveals a linear correlation between PM_2.5_ exposure and the risk of 10-year ASCVD, with China-PAR scores increasing as PM_2.5_ concentrations increasing (*p* for nonlinear = 0.656) (Figure 1).

**Table 2:** Association of PM_2.5_ exposure levels with China-PAR scores.

Three-year average PM_2.5_ exposure	*β* (95% CI)			
	Model 1^a^	*p*-value	Model 2^b^	*p*-value
Continuous variable				
Each 5 μg/m^3^ of elevation	2.12 (1.40, 2.84)	<0.001	3.25 (2.60, 3.89)	<0.001
Categorical variables (tertiles)				
Low concentration	0 (reference)		0 (reference)	
Medium concentration	2.57 (2.15, 2.99)	<0.001	1.44 (1.07, 1.82)	<0.001
High concentration	2.33 (1.72, 2.94)	<0.001	3.13 (2.58, 3.67)	<0.001
*p for trend* ^†^	<0.001		<0.001	

^Note:^ *β*, regression coefficient; CI, confidence interval; PM_2.5_, fine particulate matter; China-PAR, risk prediction of atherosclerotic cardiovascular disease in China.

*β* was obtained by fitting a linear mixed-effects model, with PM_2.5_ per 5 μg/m^3^ of elevation, using district and county as random-effects terms.

a Model 1: unadjusted model.

b Model 2: adjusted for marital status, education level, drinking status, and per capita disposable income.

^†^Trend *p*-values were obtained by coding PM_2.5_ exposure concentrations as 1, 2, and 3 in order of tertile rank and fitting the codes as measures.

### 3.3 Subgroup analyses

**Figure 2:**
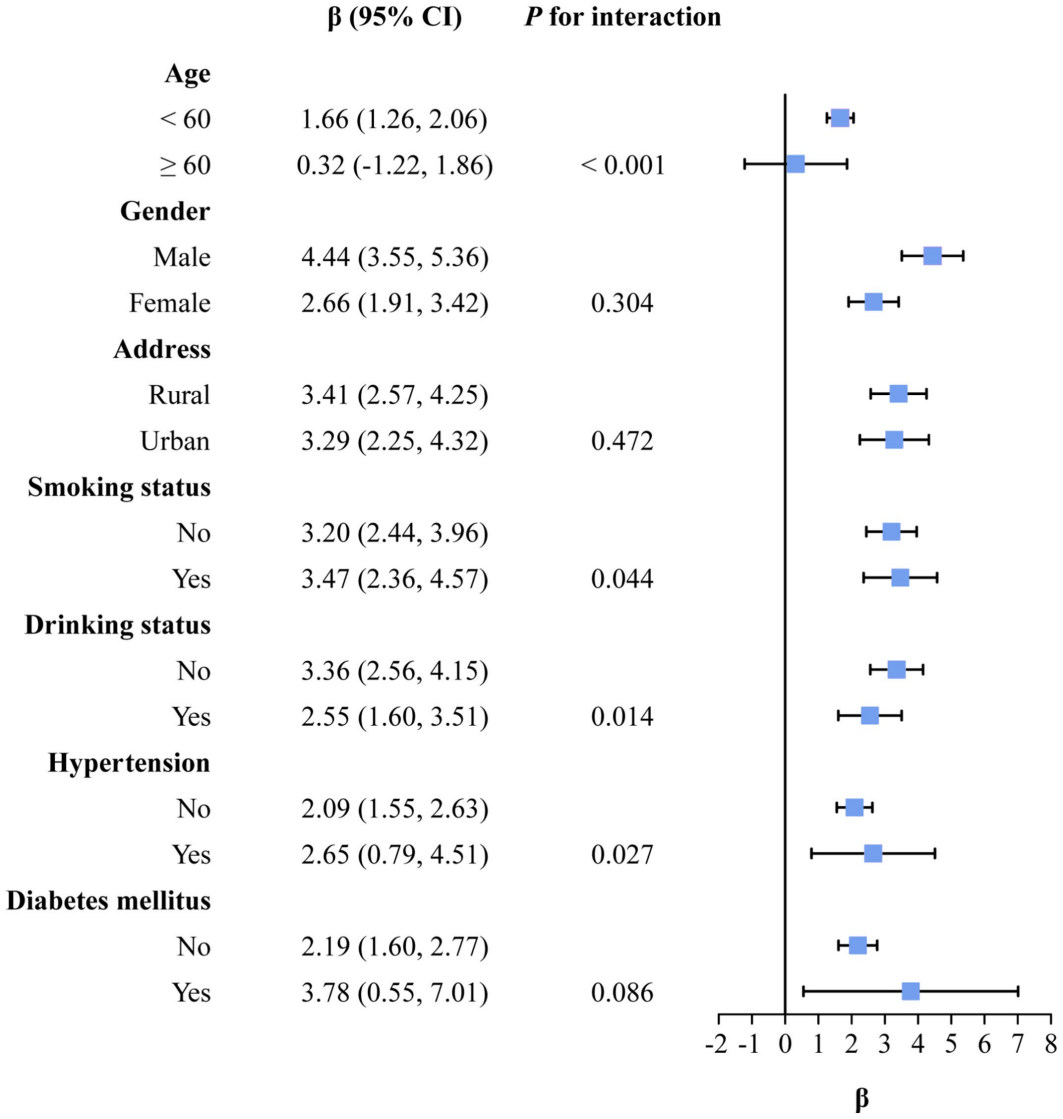
Association of PM_2.5_ exposure levels with China-PAR scores in stratified analyses. *β*, regression coefficient; CI, confidence interval; PM_2.5_, fine particulate matter; China-PAR, risk prediction of atherosclerotic cardiovascular disease in China. *β* was obtained by fitting a linear mixed-effects model, with PM_2.5_ per 5 μg/m^3^ of elevation, using district as the random-effects term. All models were adjusted for marital status, education level, drinking status, and disposable income per capita.

Figure 2 illustrates the results of stratified analysis. The stronger association was showed for PM_2.5_ exposure and 10-year ASCVD risk among participants aged under 60 years (*p*_interaction_ less than 0.05). And the detrimental effect of exposure to PM_2.5_ on ASCVD was stronger in those who were smokers, who did not drink alcohol and who had hypertension (*p*_interaction_ less than 0.05). Supplementary Figure S1 shows the exposure-response relationships of the subgroups.

### 3.4 Mediation analysis

**Figure 3:**
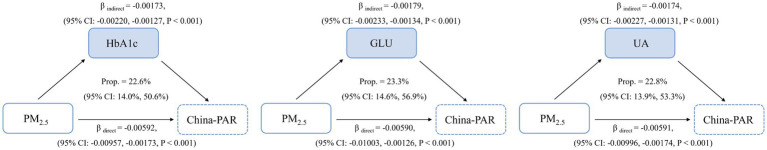
Mediation effects of routine blood, blood biochemistry, and urinalysis indicators in the association of PM_2.5_ exposure with China-PAR scores. All models were adjusted for marital status, education level, drinking status, and per capita disposable income. PM_2.5_, fine particulate matter; China-PAR, risk prediction of atherosclerotic cardiovascular disease in China; CI, confidence interval; HbA1c, glycosylated hemoglobin; GLU, fasting blood glucose; UA, uric acid.

**Figure 4:**
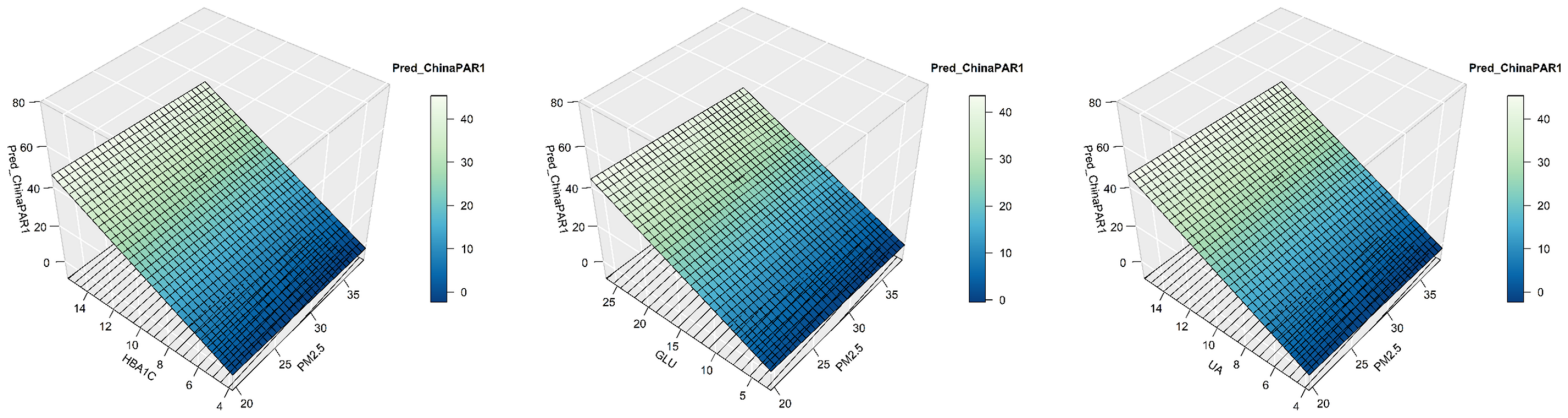
Association of PM_2.5_ exposure, mediation variables, and China-PAR scores. PM_2.5_, fine particulate matter; China-PAR, risk prediction of atherosclerotic cardiovascular disease in China; HbA1c, glycosylated hemoglobin; GLU, fasting blood glucose; UA, uric acid.

The mediation analyses suggested that the correlation between PM_2.5_ exposure and 10-year ASCVD risk was mediated by HbA1c, GLU and UA with the proportion of mediation effect ranging from 22.6 to 23.3% (Figures 3, 4 and Supplementary Table S6). Specifically, the association of exposure to PM_2.5_ with 10-year risk of ASCVD was 22.6% (95%CI: 14.0, 50.6%), 23.3% (95%CI: 14.6, 56.9%) and 22.8% (95%CI: 13.9, 53.3%), mediated by HbA1c, GLU and UA.

### 3.5 Sensitivity analysis

No substantially changed in the results of the sensitivity analysis. For each 5 μg/m^3^ rising in 1-year PM_2.5_ exposure, the China-PAR risk score increased by an average of 3.24 points (*β* = 3.24, 95% CI: 2.50, 3.98) after adjusting for covariates. Dividing the 1-year PM_2.5_ exposure into three levels, the results show that participants with medium and high PM_2.5_ exposure levels have higher 10-year ASCVD risk, with β as 1.61 (95% CI: 1.19, 2.03) and 2.43 (95% CI: 1.94, 2.93), respectively (Supplementary Table S7). For each 5 μg/m^3^ increase in 5-year PM_2.5_ exposure, the China-PAR risk score increased by an average of 2.84 points (*β* = 2.84, 95% CI: 2.27, 3.41) after adjusting for covariates. Dividing the 5-year PM_2.5_ exposure into three levels, the results show that participants with medium and high PM_2.5_ exposure levels have higher risk of 10-year ASCVD, with β as 1.29 (95% CI: 0.92, 1.66) and 3.03 (95% CI: 2.48, 3.58), respectively (Supplementary Table S8). After additional corrections for dietary score, for each 5 μg/m^3^ increase in PM_2.5_ exposure, the China-PAR risk score increased by an average of 2.35 points (*β* = 2.35, 95% CI: 1.63, 3.08) (Supplementary Table S9). After additional adjustments for CO, NO_2_, O_3_, SO_2_ and PM_1_, for each 5 μg/m^3^ increase in PM_2.5_ exposure, the China-PAR risk score increased by an average of 3.27 points (*β* = 3.27, 95% CI: 1.62, 4.92) (Supplementary Table S10).

## 4 Discussion

In current study, we explored the relationship of PM_2.5_ exposure with 10-year risk of ASCVD utilizing LME model and examined potential effect modifiers. Our results suggested that PM_2.5_ exposure may elevate 10-year risk of ASCVD after adjusting covariates. Subgroup analysis indicated that age, smoking status, drinking status, and hypertension were potential effect modifiers of this association. Additionally, mediation effects of HbA1c, GLU and UA were found in the correlation between PM_2.5_ exposure and 10-year risk of ASCVD.

Our study indicated that PM_2.5_ exposure is correlated with a greater 10-year risk of ASCVD, with higher exposure levels having a stronger effect. Our findings are to some extent consistent with previous studies. For example, a multiethnic cohort study in China explored the correlation of exposure to PM_2.5_ and its components with China-PAR scores, indicating that there is a positive relationship of long-term exposure to PM_2.5_ and its components with an increased 10-year ASCVD risk (39). Another large-scale cross-sectional study in rural Henan Province, China, revealed that long-term exposure to PM_2.5_ and its constituents is associated with the risen ASCVD risk (40). And a study from China-PAR project revealed a significant joint impact of ASCVD risk stratification and PM_2.5_ exposure on ASCVD, underscoring the possible health advantages of decreasing PM_2.5_ levels among the Chinese population, particularly for individuals at high ASCVD risk (41). However, unlike these studies, we did not use categorical variables to classify the outcome into high and low risk groups for 10-year risk of ASCVD. Instead, we used continuous variables to assess the outcome. This method of continuous variables provides a more detailed risk assessment as it allows us to consider the entire range of risk scores, rather than just dividing them into two extreme categories. Additionally, our study population was from Fujian Province, China, which broadens the applicability of the finding.

This study identified that age, smoking status, drinking status, and hypertension modify the relationship of PM_2.5_ exposure with the risk of 10-year ASCVD. A study form UK Biobank showed that the risk of air pollution exposure in the development of cardiometabolic multimorbidity, including type 2 diabetes, ischemic heart disease, and stroke, was higher among older, male, excessive alcohol intake, and lower socioeconomic status individuals (42). A Canadian cohort study demonstrated that long-term PM_2.5_ exposure is more strongly associated with the risk of cardiovascular death among regular exercisers, smokers, and those with lower household incomes (43). A Korean study showed that air pollution exposure and CVDs risk are more strongly associated in women, hypertensive patients, and urban residents (44). A cohort study from the United States found that the risk of PM_2.5_-related cardiovascular hospitalizations differs across age, educational levels, access to healthcare services, and overall level of deprivation in the community (45). A systematic review and meta-analysis also observed inconsistent differences in gender, smoking status and socio-economically deprived populations (46). The heterogeneity of our findings compared to previous studies may be attributed to the distinct demographic characteristics of the study population and the exposure windows. In addition, our study provides more possible effect modifiers to be explored, including individual demographic characteristics and lifestyle. The findings suggest that effective preventive measures and health promotion measures should be adopted in the future for susceptible populations.

HbA1c, GLU may partially mediate the association of PM_2.5_ and China-PAR scores, which suggests to some extent that changes in blood glucose and lipids may influence the development of ASCVD. GLU primarily measures blood sugar levels after an overnight fast, while HbA1c determines the average blood glucose level over an extended period. Previous researches have demonstrated that hyperglycemia is a risk factors for cardiovascular diseases (47). A Chinese prospective cohort study has indicated that normalizing blood glucose in prediabetes can significantly reduce CVDs risk and all-cause mortality risk (48). PM_2.5_ exposure may trigger inflammatory responses and oxidative stress, impairs insulin response pathways in the liver, skeletal muscle, and adipose tissue, worsens hyperglycaemia (49–51). Notably, elevated fasting blood glucose and HbA1c are both recognized as major cardiovascular risk factors for the progression of atherosclerosis and cardiovascular mortality (52). Specifically, hyperglycemia can activate immune and inflammatory responses, accelerate the progression of atherosclerosis, induce endothelial dysfunction and promote a prothrombotic state, thereby increasing the risk of cardiovascular disease (53, 54). Thus, preventing diabetes may be able to reduce 10-year ASCVD risk, and our findings provide new evidence to support this idea.

In addition, we also found that UA may partially mediate the association of PM_2.5_ and China-PAR scores. A recent representative cohort from southwestern China also explored the potential mediation of serum UA on the relationship of exposure to PM_2.5_ and metabolic syndrome, with serum uric acid mediating 13.6% of the association (55). Metabolic syndrome, characterized by concurrent metabolic dysfunctions including dyslipidemia, hypertension, and abdominal obesity, is recognized as a precursor to type 2 diabetes and CVDs (56), and thus, to some extent, supports the findings of the present study as well. Therefore, it is suggested that PM_2.5_ exposure may cause changes in blood uric acid, which in turn cause alterations in the metabolic syndrome, ultimately leading to the development of ASCVD, and subsequent studies could explore the presence of this pathway through multiple mediator analyses.

We recognize the following limitations. First, cross-sectional design does not validate causality. However, we used 1-, 3-, and 5-year average PM_2.5_ exposure concentrations and excluded subjects with CVDs at baseline to bolster the credibility of our time series of exposure outcomes. Large-scale, large population-based prospective cohort studies that provide more individual health information are needed to further explore this in the future. Second, due to the lack of precise individual exposure measurements, we obtained exposure data by matching based on residential address. This approach, however, does not facilitate precise exposure information, potentially resulting in nondifferential misclassification bias and consequently, an underestimation of risk. Third, air pollution composition is complex and dynamically changing, but we only assessed PM_2.5_ exposure and the results may have been influenced by other exposures. Fourth, the history of hypertension and diabetes mellitus in the survey was derived from self-reporting by the study participants, which may be subjective bias. Fifth, unmeasured confounders may have influenced the relationship between exposure and outcome. Sixth, the screening indicators included in the analysis of mediating factors are preliminary and not comprehensive enough, and different indicators may interact with each other. Therefore, in the future, more detailed studies are needed to explore the possible interactions between different indicators. Finally, the simplified model assumptions of traditional mediation analysis we employ struggle to capture the complex interactions and nonlinear mediating mechanisms among biomarkers. In future studies, incorporating additional mediating variables and employing high-dimensional mediation analysis methods could be considered.

## 5 Conclusion

In conclusion, long-term exposure to PM_2.5_ was linked to elevated high 10-year ASCVD risk, with this correlation being partly mediated by HbA1c, GLU and UA. Age, smoking status, drinking status and hypertension were recognized as effect modifiers for the association of exposure to PM_2.5_ and 10-year ASCVD risk. The latest research data show a significant reduction in the burden of disease linked to PM_2.5_ following the implementation of China’s clean air actions (57), a result that clearly demonstrates the benefits that residents can derive from the clean air actions and underscores the importance of continuing to push forward with air quality improvement measures. Therefore, continued attention to the environmental impact on cardiovascular health and effective measures to control air pollution are important for effective health promotion. At the same time, our findings provide ideas for the practical application of measures to prevent the occurrence of CVDs. And the potential for cardiovascular damage from air pollution may be reduced by controlling mediators (e.g., controlling blood glucose, etc.), providing a basis for promoting disease prevention.

## Data Availability

The raw data supporting the conclusions of this article will be made available by the authors without undue reservation.
