# Supplementary material for: Association of PM2.5 exposure with 10-year atherosclerotic cardiovascular disease risk: a mediation analysis of blood and urinary biomarkers
Source: Front Public Health. 2026 Apr 10;14:1799605. doi: 10.3389/fpubh.2026.1799605 (PMC13106173; doi:10.3389/fpubh.2026.1799605)
Supplement: Supplementary file 1 [file Data_Sheet_1.docx]

**Supplementary material**

**1. Supplementary methods**

**1.1 Cardiovascular disease and its risk factor surveillance in Chinese residents**

The study survey used stratified multi-stage random sampling to establish monitoring points based on nationally and provincially representative sampling points, with two townships (streets) per monitoring point, three village (neighborhood) committees every township (street), and 1,200 people aged 18 years and older who had been living in the area for more than six months every village (neighborhood) committee, and 1,200 people per monitoring point, according to gender and age group. The number of people in each monitoring point is 1200 by gender and age group. The on-site survey included: registration of basic information (name, gender, address and other basic information), blood pressure, blood sampling, urine retention, questionnaire, weight, body composition measurements, ankle-brachial index, electrocardiogram and ultrasound.

**1.2** **Assessment of PM_2.5_ exposure**

CHAP dataset was produced by using artificial intelligence techniques, taking into account the spatial and temporal heterogeneous characteristics of air pollution and produced from big data (e.g., ground-based observations, satellite remote sensing products, atmospheric reanalysis and model simulation information, etc.). PM_2.5_ dataset (China High PM_2.5_), one of the dataset from the CHAP dataset, utilizes the long-term, high temporal and spatial resolution aerosol optical depth (AOD) generated by the MODIS Multi-Angle implementation of Atmospheric Correction (MAIAC) algorithm, and the Space-Time Extra-Trees (STET) model, which combines the temporal and spatial information with the information on the surface conditions and human activities, to estimate the PM_2.5_ concentration at a resolution of 1 km over the region of China. The accuracy of this dataset was reliable, with a ten-fold cross-validation coefficient of determination R^2^ of 0.92, a root mean square error RMSE of 10.76 µg/m^3^, and a mean absolute error of 6.32 µg/m^3^. ArcGIS is a Geographic Information Systems (GIS) developed by Environmental systems research institute (ESRI). GIS is a specific and very important spatial information system, which is based on geospatial, using geographic modeling and analysis methods to provide a variety of spatial and dynamic geographic information in real time, is a kind of computer technology system for geographic research and geographic decision-making services. It is a technology that collects, stores, manages, processes, analyzes, displays and describes relevant geographic distribution data in the space of the whole or part of the Earth's surface layer (including the atmosphere) with the support of computer hardware and software systems. The residential address provided by the study subjects was geocoded using Map Location (<https://maplocation.sjfkai.com/>) to match individual latitude and longitude coordinates.

**1.3 Quality control**

**1.3.1 Quality control of questionnaires**

Formulate a unified questionnaire and instructions for filling in the questionnaire, and conduct unified training and assessment for each investigator to familiarize him/her with each question in the questionnaire and to master the questioning techniques. Conduct daily self-inspection of the questionnaire to check for errors, omissions and obvious logical errors. EpiData 3.1 software was applied to double-entry data for logical error correction and consistency testing. Surveys based on the monitoring of cardiovascular disease and its risk factors in the population also require quality controllers at the monitoring sites to check all the survey forms every day for omissions and errors, and when omissions and errors are found, the investigators and survey respondents are contacted in time for verification and correction. The person in charge of the survey team comprehensively monitors, coordinates and organizes the on-site survey. Conducting inspections of all survey work and corrects any problems promptly.

**1.3.2 Quality control of laboratory**

Personnel involved in blood specimen collection, separation, storage and transportation are strictly trained and assessed so that they are familiar with the standard procedures and precautions for various operations, and are allowed to participate in the relevant work only after passing the assessment. Laboratory conditions should be strictly inspected before the project starts, and the work can be started only after meeting the standards. The specimen storage process shall be taken care of by specialized personnel, and the temperature of the refrigerator and the status of the specimen shall be registered every day. The transportation process shall be escorted by a person, and sufficient amount of refrigerant shall be placed according to the requirements. External quality control of the laboratory: The determination of lipids, blood glucose and other indicators must pass the quality supervision and certification of the relevant authorities. Internal quality control of the laboratory: Laboratory quality control using the quality control chart method During specimen testing, each batch of specimens is quality controlled by determining two quality control levels 1 and 2 for the relevant indicators. Parallel sample testing randomly selected 1% of the specimens for parallel sample determination, to determine the repeatability of the two measurements, and statistical tests. Manual monitoring, in the operation process, if the testing personnel found that the measured value of an indicator is abnormal, the batch of samples should also be re-tested; after the daily testing is completed and the testing data is entered into the database, an experienced technician carefully reads the test results for the day and, if any abnormality is detected, the test is re-tested on the following day.

**1.4 China-PAR risk scores**

The China-PAR risk assessment model included^[1]^: gender, age, current residence (urban or rural), geographic region (north or south, bounded by the Yangtze River), waist circumference, total cholesterol (TC), high density lipoprotein cholesterol (HDL-C), systolic blood pressure (SBP) (treated or untreated), use of antihypertensive drugs (/non), diabetes mellitus (/non), smoking (/non), and cardiovascular risk. C), systolic blood pressure (SBP) (treated or untreated), use of antihypertensive medication (yes/no), diabetes mellitus (yes/no), smoking (yes/no), family history of cardiovascular disease (yes/no), and the interaction of age with various risk factors.

Table S1

Coefficients of the equations of the prediction model for predicting ASCVD risk grouped by gender.

|  | Male | | Female |
| --- | --- | --- | --- |
| Ln (age), y | | 31.97 | 24.87 |
| Ln (treated SBP), mmHg | | 27.39 | 20.71 |
| Ln (untreated SBP), mmHg | | 26.15 | 19.98 |
| Ln (TC), mg/dL | | 0.62 | 0.06 |
| Ln (HDL-C), mg/dL | | -0.69 | -0.22 |
| Ln (WC), cm | | -0.71 | 1.48 |
| Smoking (1=yes, 0=no) | | 3.96 | 0.49 |
| Diabetes mellitus (1=yes, 0=no) | | 0.36 | 0.57 |
| Geographic area (1=north of China, 0=south of China) | | 0.48 | 0.54 |
| Address (1=urban, 0=rural) | | -0.16 | NA |
| Family history of ASCVD (1=yes, 0=no) | | 6.22 | NA |
| Ln (age)*Ln (treated SBP) | | -6.02 | -4.53 |
| Ln (age)*Ln (untreated SBP) | | -5.73 | -4.36 |
| Ln (age)*smoking status | | -0.94 | NA |
| Ln (age)*family history of ASCVD | | -1.53 | NA |

*Abbreviations: ASCVD, atherosclerotic cardiovascular disease; China-PAR, China Prediction of Atherosclerotic Cardiovascular Disease Risk; SBP, systolic blood pressure; TC, total cholesterol; HDL-C, high-density lipoprotein cholesterol; WC, waist circumference; Ln, natural logarithm; N/A, this covariate is not included in the equation.

Next, the values assigned to each variable were multiplied by the coefficients by sex, and the sum of the coefficients × values columns was used to calculate the sum of the individuals in each sex group, which is displayed as the "Individual Sum".

The formula for calculating the 10-year predicted risk of developing ASCVD is as follows:

$$1-S_{10}^{e^{(lndX^{'}B-MeanX'B)}}$$

S_10_ is the 10-year survival rate for ASCVD, which was 0.9707 for males and 0.9851 for females. lndX'B is the gender-specific "coefficient x value" overall sum, and MeanX'B is the gender-specific "coefficient x value" overall mean sum, which was 140.68 for males and 117.26 for females. The overall mean sum is 140.68 for males and 117.26 for females.

Example 1: Male, 60 years old, untreated systolic blood pressure 130 mmHg, total cholesterol 210 mg/dL, high dense lipoprotein cholesterol 55 mg/dL, waist circumference 80 cm, nonsmoker, diabetes mellitus, living in an urban area of northern China, no family history of ASCVD.

Table S2

Example table for calculating China-PAR risk score for male.

|  | Male | | | |
| --- | --- | --- | --- | --- |
|  | β | Example value | Ln (Continuous variable) | β*Ln (Continuous variable) |
| Ln (age), y | 31.97 | 60.00 | 4.09 | 130.88 |
| Ln (treated SBP), mmHg | 27.39 | - | - | - |
| Ln (untreatedSBP), mmHg | 26.15 | 130.00 | 4.87 | 127.28 |
| Ln (total cholesterol), mg/dL | 0.62 | 210.00 | 5.35 | 3.32 |
| Ln (HDL-C), mg/dL | -0.69 | 55.00 | 4.01 | -2.78 |
| Ln (WC), cm | -0.71 | 80.00 | 4.38 | -3.12 |
| Smoking status (1=yes, 0=no) | 3.96 | 0 | 0.00 | 0.00 |
| Diabetes mellitus (1=yes, 0=no) | 0.36 | 1 | 1.00 | 0.36 |
| Geographic area (1=north of China, 0=south of China) | 0.48 | 1 | 1.00 | 0.48 |
| Address (1=urban, 0=rural) | -0.16 | 1 | 1.00 | -0.16 |
| Family history of ASCVD (1=yes, 0=no) | 6.22 | 0 | 0.00 | 0.00 |
| Ln (age)*Ln (treated SBP) | -6.02 | - | - | - |
| Ln (age)*Ln (untreated SBP) | -5.73 | 190.00 | 19.93 | -114.21 |
| Ln (age)*smoking status | -0.94 | 0 | 0.00 | 0.00 |
| Ln (age)*family history of ASCVD | -1.53 | 0 | 0.00 | 0.00 |
| ASCVD |  |  |  |  |
| Individual total | N/A |  |  | 142.04 |
| Average total (β*value) | N/A |  |  | 140.68 |
| 10-year survival rate | N/A |  |  | 0.97 |
| Assessed 10-year risk (%) | N/A |  |  | 10.97 |

*Abbreviations: ASCVD, atherosclerotic cardiovascular disease; China-PAR, China Prediction of Atherosclerotic Cardiovascular Disease Risk; SBP, systolic blood pressure; TC, total cholesterol; HDL-C, high-density lipoprotein cholesterol; WC, waist circumference; Ln, natural logarithm; N/A, this covariate is not included in the equation.

Unit conversion: convert TC and HDL-C to mmol/L by multiplying by 0.0259. Individual example values: natural logarithm of continuous covariate, natural logarithm of categorical covariate is 0 or 1, and the interaction term is the product of the natural logarithm of age multiplied by the natural logarithm of the continuous covariate or multiplied by the value of the categorical covariate.

Example 2: female, 60 years old, untreated systolic blood pressure 130 mmHg, total cholesterol 210 mg/dL, high dense lipoprotein cholesterol 55 mg/dL, waist circumference 80 cm, nonsmoker, diabetes mellitus, living in an urban area in northern China, no family history of ASCVD.

Table S3

Example table for calculating China-PAR risk score for male.

|  | Male | | | |
| --- | --- | --- | --- | --- |
|  | β | Example value | Ln (Continuous variable) | β*Ln (Continuous variable) |
| Ln (age), y | 24.87 | 60.00 | 4.09 | 101.84 |
| Ln (treated SBP), mmHg | 20.71 | - | - | - |
| Ln (untreatedSBP), mmHg | 19.98 | 130.00 | 4.87 | 97.26 |
| Ln (total cholesterol), mg/dL | 0.06 | 210.00 | 5.35 | 0.31 |
| Ln (HDL-C), mg/dL | -0.22 | 55.00 | 4.01 | -0.87 |
| Ln (WC), cm | 1.48 | 80.00 | 4.38 | 6.46 |
| Smoking status (1=yes, 0=no) | 0.49 | 0.00 | 0.00 | 0.00 |
| Diabetes mellitus (1=yes, 0=no) | 0.57 | 1.00 | 1.00 | 0.57 |
| Geographic area (1=north of China, 0=south of China) | 0.54 | 1.00 | 1.00 | 0.54 |
| Address (1=urban, 0=rural) | - | 1.00 | 1.00 | - |
| Family history of ASCVD (1=yes, 0=no) | - | 0.00 | 0.00 | - |
| Ln (age)*Ln (treated SBP) | -4.53 | - | - | - |
| Ln (age)*Ln (untreated SBP) | -4.36 | 190.00 | 19.93 | -86.90 |
| Ln (age)*smoking status | - | 0.00 | 0.00 | - |

Table S3 (Continued)

|  | Male | | | |
| --- | --- | --- | --- | --- |
|  | β | Example value | Ln (Continuous variable) | β*Ln (Continuous variable) |
| Ln (age)*family history of ASCVD | - | 0.00 | 0.00 | - |
| ASCVD |  |  |  |  |
| Individual total | N/A |  |  | 119.22 |
| Average total (β*value) | N/A |  |  | 117.26 |
| 10-year survival rate | N/A |  |  | 0.98 |
| Assessed 10-year risk (%) | N/A |  |  | 10.14 |

*Abbreviations: ASCVD, atherosclerotic cardiovascular disease; China-PAR, China Prediction of Atherosclerotic Cardiovascular Disease Risk; SBP, systolic blood pressure; TC, total cholesterol; HDL-C, high-density lipoprotein cholesterol; WC, waist circumference; Ln, natural logarithm; N/A, this covariate is not included in the equation.

Unit conversion: convert TC and HDL-C to mmol/L by multiplying by 0.0259. Individual example values: natural logarithm of continuous covariate, natural logarithm of categorical covariate is 0 or 1, and the interaction term is the product of the natural logarithm of age multiplied by the natural logarithm of the continuous covariate or multiplied by the value of the categorical covariate.

**Reference**

[1] Yang X, Li J, Hu D, et al. Predicting the 10-Year Risks of Atherosclerotic Cardiovascular Disease in Chinese Population: The China-PAR Project (Prediction for ASCVD Risk in China) [J]. Circulation, 2016, 134(19): 1430-40.

Table S4

Descriptive statistic of the mediating variables.

| Mediating variables | median [IQR] |
| --- | --- |
| Leucocyte (10^9^ /L) | 6.39 [5.42, 7.54] |
| Erythrocyte (10^12^ /L) | 4.79 [4.44, 5.19] |
| Hemoglobin (g /L) | 143 [132, 155] |
| Neutrophil (10^9^ /L) | 3.61 [2.89, 4.46] |
| Platelet (10^9^ /L) | 255 [218, 295] |
| Glycosylated hemoglobin (%) | 5.50 [5.20, 5.80] |
| Fasting blood glucose (mmol/L) | 5.18 [4.75, 5.66] |
| Triglyceride (mmol/L) | 1.14 [0.79, 1.71] |
| Low-density lipoprotein (LDL) cholesterol (mmol/L) | 2.99 [2.46, 3.60] |
| Creatinine (μmoI/L) | 73 [62, 86] |
| Uric acid (μmoI/L) | 5.50 [5.20, 5.80] |
| Potassium (mmoI/L) | 4.20 [3.98, 4.44] |
| Urinary creatinine (μmoI/L) | 13.637 [9.039, 17.215] |
| Urine microalbumin (μg/mL) | 7 [3, 15] |

Table S5

Characteristics of the study participants and PM_2.5_ stratified by China-PAR score.

| Variables | China-PAR scores | | | *P-*value |
| --- | --- | --- | --- | --- |
|  | Low risk  (n=6179) | Medium risk  (n=1166) | High risk  (n=2009) |  |
| **Age (median [IQR]), years** | 34.00 [25.00, 43.00] | 55.00 [51.00, 60.00] | 70.00 [63.00, 77.00] | ＜0.001 |
| **Gender (%)** |  |  |  | ＜0.001 |
| Male | 2761 (44.7) | 645 (55.3) | 1190 (59.2) |  |
| Female | 3418 (55.3) | 521 (44.7) | 819 (40.8) |  |
| **Marital status, n (%)** |  |  |  | ＜0.001 |
| Married | 4012 (64.9) | 1081 (92.7) | 1639 (81.6) |  |
| Unmarried/Others | 2167 (35.1) | 85 (7.3) | 370 (18.4) |  |
| **Hypertension, n (%)** |  |  |  | ＜0.001 |
| No | 6083 (98.4) | 953 (81.7) | 1084 (54.0) |  |
| Yes | 96 (1.6) | 213 (18.3) | 925 (46.0) |  |

Table S5 (Continued)

| Variables | China-PAR scores | | | *P-*value |
| --- | --- | --- | --- | --- |
|  | Low risk  (n=6179) | Medium risk  (n=1166) | High risk  (n=2009) |  |
| **Drinking status, n (%)** |  |  |  | ＜0.001 |
| No | 4170 (67.5) | 776 (66.6) | 1478 (73.6) |  |
| Yes | 2009 (32.5) | 390 (33.4) | 531 (26.4) |  |
| **Smoking status, n (%)** |  |  |  | ＜0.001 |
| No | 5087 (82.3) | 777 (66.6) | 1368 (68.1) |  |
| Yes | 1092 (17.7) | 389 (33.4) | 641 (31.9) |  |
| **Diabetes mellitus, n (%)** |  |  |  | ＜0.001 |
| No | 6144 (99.4) | 1122 (96.2) | 1762 (87.7) |  |
| Yes | 35 (0.6) | 44 (3.8) | 247 (12.3) |  |
| **Address, n (%)** |  |  |  | 0.001 |
| Rural | 3784 (61.2) | 772 (66.2) | 1297 (64.6) |  |
| Urban | 2395 (38.8) | 394 (33.8) | 712 (35.4) |  |

Table S5 (Continued)

| Variables | China-PAR scores | | | *P-*value |
| --- | --- | --- | --- | --- |
|  | Low risk  (n=6179) | Medium risk  (n=1166) | High risk  (n=2009) |  |
| **Family history of cardiovascular disease, n (%)** |  |  |  | ＜0.001 |
| No | 6040 (97.8) | 1102 (94.5) | 1946 (96.9) |  |
| Yes | 139 (2.2) | 64 (5.5) | 63 (3.1) |  |
| **Antihypertension drugs intake, n (%)** |  |  |  | ＜0.001 |
| No | 6114 (98.9) | 976 (83.7) | 1144 (56.9) |  |
| Yes | 65 (1.1) | 190 (16.3) | 865 (43.1) |  |
| **Education, n (%)** |  |  |  | ＜0.001 |
| Illiteracy | 219 (3.5) | 251 (21.5) | 690 (34.3) |  |
| Primary school | 594 (9.6) | 326 (28.0) | 672 (33.4) |  |
| Junior high school | 1476 (23.9) | 306 (26.2) | 393 (19.6) |  |
| High school/trade school | 1368 (22.1) | 183 (15.7) | 183 (9.1) |  |
| Junior college or above | 2522 (40.8) | 100 (8.6) | 71 (3.5) |  |

Table S5 (Continued)

| Variables | China-PAR scores | | | *P-*value |
| --- | --- | --- | --- | --- |
|  | Low risk  (n=6179) | Medium risk  (n=1166) | High risk  (n=2009) |  |
| **Per capita disposable income, n (%)** |  |  |  | ＜0.001 |
| [0, 10000) | 386 (6.2) | 166 (14.2) | 562 (28.0) |  |
| [10000, 20000) | 1229 (19.9) | 272 (23.3) | 510 (25.4) |  |
| [20000, 30000) | 1445 (23.4) | 296 (25.4) | 401 (20.0) |  |
| [30000, 50000) | 1378 (22.3) | 193 (16.6) | 274 (13.6) |  |
| [50000, 100000) | 1132 (18.3) | 153 (13.1) | 182 (9.1) |  |
| [100000, Inf) | 609 (9.9) | 86 (7.4) | 80 (4.0) |  |
| **SBP (median [IQR]), mm Hg** | 120.00 [111.00, 129.67] | 136.67 [127.00, 147.00] | 150.67 [137.33, 163.67] | ＜0.001 |
| **TC (median [IQR]), mmol/L** | 4.65 [4.10, 5.31] | 5.23 [4.60, 5.97] | 5.20 [4.45, 5.87] | ＜0.001 |
| **HDL-C (median [IQR]), mmol/L** | 1.41 [1.21, 1.62] | 1.35 [1.16, 1.59] | 1.33 [1.14, 1.54] | ＜0.001 |
| **WC (median [IQR]), cm** | 77.00 [70.00, 84.20] | 84.00 [77.60, 90.00] | 84.60 [78.00, 91.00] | ＜0.001 |

Table S5 (Continued)

| Variables | China-PAR scores | | | *P-*value |
| --- | --- | --- | --- | --- |
|  | Low risk  (n=6179) | Medium risk  (n=1166) | High risk  (n=2009) |  |
| **BMI (median [IQR]), kg/m^2^** | 22.93 [20.69, 25.46] | 24.42 [22.43, 26.93] | 24.19 [22.08, 26.57] | ＜0.001 |
| **One-year average PM_2.5_ exposure concentration**  **(median [IQR]), μg/m^3^** | 21.20 [19.90, 22.80] | 21.20 [20.10, 22.80] | 21.20 [20.10, 22.10] | 0.267 |
| **Three-year average PM_2.5_ exposure concentration**  **(median [IQR]), μg/m^3^** | 23.40 [22.17, 25.60] | 23.23 [22.37, 25.53] | 23.20 [22.43, 24.50] | 0.77 |
| **Five-year average PM_2.5_ exposure concentration**  **(median [IQR]), μg/m^3^** | 25.40 [23.90, 28.20] | 25.40 [24.14, 27.68] | 24.88 [24.10, 26.36] | 0.928 |
| **China PAR scores (median [IQR])** | 0.61 [0.14, 1.83] | 7.17 [5.97, 8.51] | 17.03 [13.00, 22.42] | ＜0.001 |

*Data are the median [Inter-quartile range] for continuous variables and number (percentage) for categorical variables.

Abbreviations: SBP, systolic blood pressure; TC, total cholesterol; HDL-C, high-density lipoprotein cholesterol; WC, waist circumference; BMI, body mass index; PM_2.5_, fine particulate matter; China-PAR, risk prediction of atherosclerotic cardiovascular disease in China.

Table S6

Mediating effects in the association between PM_2.5_ exposure and China-PAR scores.

| Mediation variables | Total effect | | Average causal mediation effect (ACME) | | Average direct effect (ADE) | | Mediation percentage,  % (*95% CI*) |
| --- | --- | --- | --- | --- | --- | --- | --- |
|  | Coefficient (*95% CI*) | *P*-value | Coefficient (*95% CI*) | *P*-value | Coefficient (*95% CI*) | *P*-value |  |
| Leucocyte | -0.00778  (-0.01191, -0.00350) | ＜0.001 | 0.00001  (-0.00004, 0.00183) | 0.756 | -0.00779  (-0.01270, -0.00385) | ＜0.001 | - |
| Erythrocyte | 0.19851  (0.14817, 0.25571) | ＜0.001 | 0.00015  (-0.00022, 0.00128) | 0.392 | 0.19836  (0.14754, 0.25579) | ＜0.001 | 0.1 (-0.1, 0.7) |
| Hemoglobin | -0.00776  (-0.01191, -0.00350) | ＜0.001 | 0.00061  (0.00032, 0.00094) | ＜0.001 | -0.00837  (-0.01255, -0.00405) | ＜0.001 | - |
| Neutrophil | -0.00778  (-0.01194, -0.00351) | ＜0.001 | 0.00002  (-0.00006, 0.00050) | 0.616 | -0.00781  (-0.01197, -0.00359) | ＜0.001 | - |
| Platelet | -0.00777  (-0.01192, -0.00350) | ＜0.001 | 0.00008  (-0.00005, 0.00027) | 0.252 | -0.00785  (-0.01194, -0.00354) | ＜0.001 | - |
| HbA1c | -0.00765  (-0.01131, -0.00369) | ＜0.001 | -0.00173  (-0.00220, -0.00127) | ＜0.001 | -0.00592  (-0.00957, -0.00173) | ＜0.001 | 22.6 (14.0, 50.6) |
| GLU | -0.00768  (-0.01183, -0.00311) | ＜0.001 | -0.00179  (-0.00233, -0.00134) | ＜0.001 | -0.00590  (-0.01003, -0.00126) | 0.008 | 23.3 (14.6, 56.9) |
| Triglyceride | -0.00687  (-0.01079, -0.00295) | ＜0.001 | -0.00015  (-0.00053, 0.00019) | 0.436 | -0.00672  (-0.01075, -0.00279) | ＜0.001 | 2.1 (-3.6, 8.9) |
| LDL-C | -0.00760  (-0.01188, -0.00376) | ＜0.001 | -0.00017  (-0.00054, 0.00020) | 0.364 | -0.00743  (-0.01174, -0.00352) | ＜0.001 | 2.2 (-3.3, 8.8) |

Table S6 (Continued)

| Mediation variables | Total effect | | Indirect effect | | Direct effect | | Mediation percentage,  % (*95% CI*) |
| --- | --- | --- | --- | --- | --- | --- | --- |
|  | Coefficient (*95% CI*) | *P*-value | Coefficient (*95% CI*) | *P*-value | Coefficient (*95% CI*) | *P*-value |  |
| Creatinine | -0.00770  (-0.01159, -0.00307) | ＜0.001 | 0.00011  (-0.00008, 0.00033) | 0.276 | -0.00780  (-0.01174, -0.00321) | ＜0.001 | - |
| UA | -0.00765  (-0.01193, -0.00364) | ＜0.001 | -0.00174  (-0.00227, -0.00131) | ＜0.001 | -0.00591  (-0.00996, -0.00174) | ＜0.001 | 22.8 (13.9, 53.3) |
| Potassium | -0.00762  (-0.01205, -0.00365) | ＜0.001 | 0.00002  (-0.00005, 0.00010) | 0.584 | -0.00764  (-0.01208, -0.00370) | ＜0.001 | - |
| Urinary creatinine | -0.00768  (-0.01143, -0.00358) | ＜0.001 | -0.00022  (-0.00078, 0.00027) | 0.372 | -0.00745  (-0.01117, -0.00338) | ＜0.001 | 2.9 (-3.8, 13.5) |
| Urine microalbumin | -0.00775  (-0.01155, -0.00360) | ＜0.001 | 0.00022  (-0.00015, 0.00066) | 0.276 | -0.00797  (-0.01187, -0.00373) | ＜0.001 | - |

* All models were adjusted for marital status, education level, drinking status, and per capita disposable income. Abbreviations: PM_2.5_, fine particulate matter; China-PAR, risk prediction of atherosclerotic cardiovascular disease in China; CI, confidence interval; HbA1c, glycosylated hemoglobin; GLU, fasting blood glucose; LDL-C, low-density lipoprotein cholesterol; UA, uric acid.

Table S7

Association between one-year average PM_2.5_ exposure and China-PAR scores.

| One-year average PM_2.5_ exposure | β (95% CI) | | | |
| --- | --- | --- | --- | --- |
|  | Model 1^a^ | *P*-value | Model 2^b^ | *P*-value |
| **Continuous variable** |  |  |  |  |
| Each 5 μg/m^3^ of elevation | 1.253 (0.411, 2.094) | 0.004 | 3.241 (2.502, 3.980) | ＜0.001 |
| **Categorical variables (tertiles)** |  |  |  |  |
| Low concentration | 0 (reference) |  | 0 (reference) |  |
| Medium concentration | 1.659 (1.176, 2.142) | ＜0.001 | 1.612 (1.190, 2.034) | ＜0.001 |
| High concentration | 1.219 (0.651, 1.787) | ＜0.001 | 2.432 (1.935, 2.928) | ＜0.001 |
| *P for trend* ^†^ | ＜0.001 | | ＜0.001 | |

* Abbreviations: β, regression coefficient; CI, confidence interval; PM_2.5_, fine particulate matter; China-PAR, risk prediction of atherosclerotic cardiovascular disease in China.

β was obtained by fitting a linear mixed-effects model, with PM_2.5_ per 5 μg/m^3^ of elevation, using district and county as random-effects terms.

^a^ Model 1: no adjustment;

^b^ Model 2: adjusted for marital status, education level, drinking status, and per capita disposable income;

^†^ Trend P-values were obtained by coding PM_2.5_ exposure concentrations as 1, 2, and 3 in order of tertile rank and fitting the codes as measures.

Table S8

Association between five-year average PM_2.5_ exposure and China-PAR scores.

| Five-year average PM_2.5_ exposure | β (95% CI) | | | |
| --- | --- | --- | --- | --- |
|  | Model 1^a^ | *P*-value | Model 2^b^ | *P*-value |
| **Continuous variable** |  |  |  |  |
| Each 5 μg/m^3^ of elevation | 1.906 (1.269, 2.543) | ＜0.001 | 2.836 (2.265, 3.406) | ＜0.001 |
| **Categorical variables (tertiles)** |  |  |  |  |
| Low concentration | 0 (reference) |  | 0 (reference) |  |
| Medium concentration | 2.435 (2.022, 2.847) | ＜0.001 | 1.290 (0.922, 1.657) | ＜0.001 |
| High concentration | 2.430 (1.811, 3.048) | ＜0.001 | 3.029 (2.480, 3.578) | ＜0.001 |
| *P for trend* ^†^ | ＜0.001 | | ＜0.001 | |

* Abbreviations: β, regression coefficient; CI, confidence interval; PM_2.5_, fine particulate matter; China-PAR, risk prediction of atherosclerotic cardiovascular disease in China.

β was obtained by fitting a linear mixed-effects model, with PM_2.5_ per 5 μg/m^3^ of elevation, using district and county as random-effects terms.

^a^ Model 1: no adjustment;

^b^ Model 2: adjusted for marital status, education level, drinking status, and per capita disposable income;

^†^ Trend P-values were obtained by coding PM_2.5_ exposure concentrations as 1, 2, and 3 in order of tertile rank and fitting the codes as measures.

Table S9

Association of PM_2.5_ exposure levels with China-PAR scores after additional adjustment for dietary factors.

| Three-year average PM_2.5_ exposure | β (95% CI) | *P*-value |
| --- | --- | --- |
| **Continuous variable** |  |  |
| Each 5 μg/m^3^ of elevation | 2.353 (1.630, 3.075) | ＜0.001 |
| **Categorical variables (tertiles)** |  |  |
| Low concentration | 0 (reference) |  |
| Medium concentration | 1.169 (0.749, 1.589) | ＜0.001 |
| High concentration | 2.346 (1.753, 2.939) | ＜0.001 |
| *P for trend* ^†^ | ＜0.001 | |

* Abbreviations: β, regression coefficient; CI, confidence interval; PM_2.5_, fine particulate matter; China-PAR, risk prediction of atherosclerotic cardiovascular disease in China.

β was obtained by fitting a linear mixed-effects model, with PM_2.5_ per 5 μg/m^3^ of elevation, using district and county as random-effects terms.

This model adjusted for marital status, education level, drinking status, per capita disposable income and dietary score.

^†^ Trend P-values were obtained by coding PM_2.5_ exposure concentrations as 1, 2, and 3 in order of tertile rank and fitting the codes as measures.

Table S10

Association of PM_2.5_ exposure levels with China-PAR scores after additional adjustment for CO, NO_2_, O_3_, SO_2_ and PM_1_.

| Three-year average PM_2.5_ exposure | β (95% CI) | *P*-value |
| --- | --- | --- |
| **Continuous variable** |  |  |
| Each 5 μg/m^3^ of elevation | 3.269 (1.620, 4.917) | ＜0.001 |
| **Categorical variables (tertiles)** |  |  |
| Low concentration | 0 (reference) |  |
| Medium concentration | 1.087 (0.632, 1.542) | ＜0.001 |
| High concentration | 1.861 (0.938, 2.784) | ＜0.001 |
| *P for trend* ^†^ | ＜0.001 | |

* Abbreviations: β, regression coefficient; CI, confidence interval; PM_2.5_, fine particulate matter; China-PAR, risk prediction of atherosclerotic cardiovascular disease in China.

β was obtained by fitting a linear mixed-effects model, with PM_2.5_ per 5 μg/m^3^ of elevation, using district and county as random-effects terms.

This model adjusted for marital status, education level, drinking status, per capita disposable income, CO, NO_2_, O_3_, SO_2_ and PM_1_.

^†^ Trend P-values were obtained by coding PM_2.5_ exposure concentrations as 1, 2, and 3 in order of tertile rank and fitting the codes as measures.


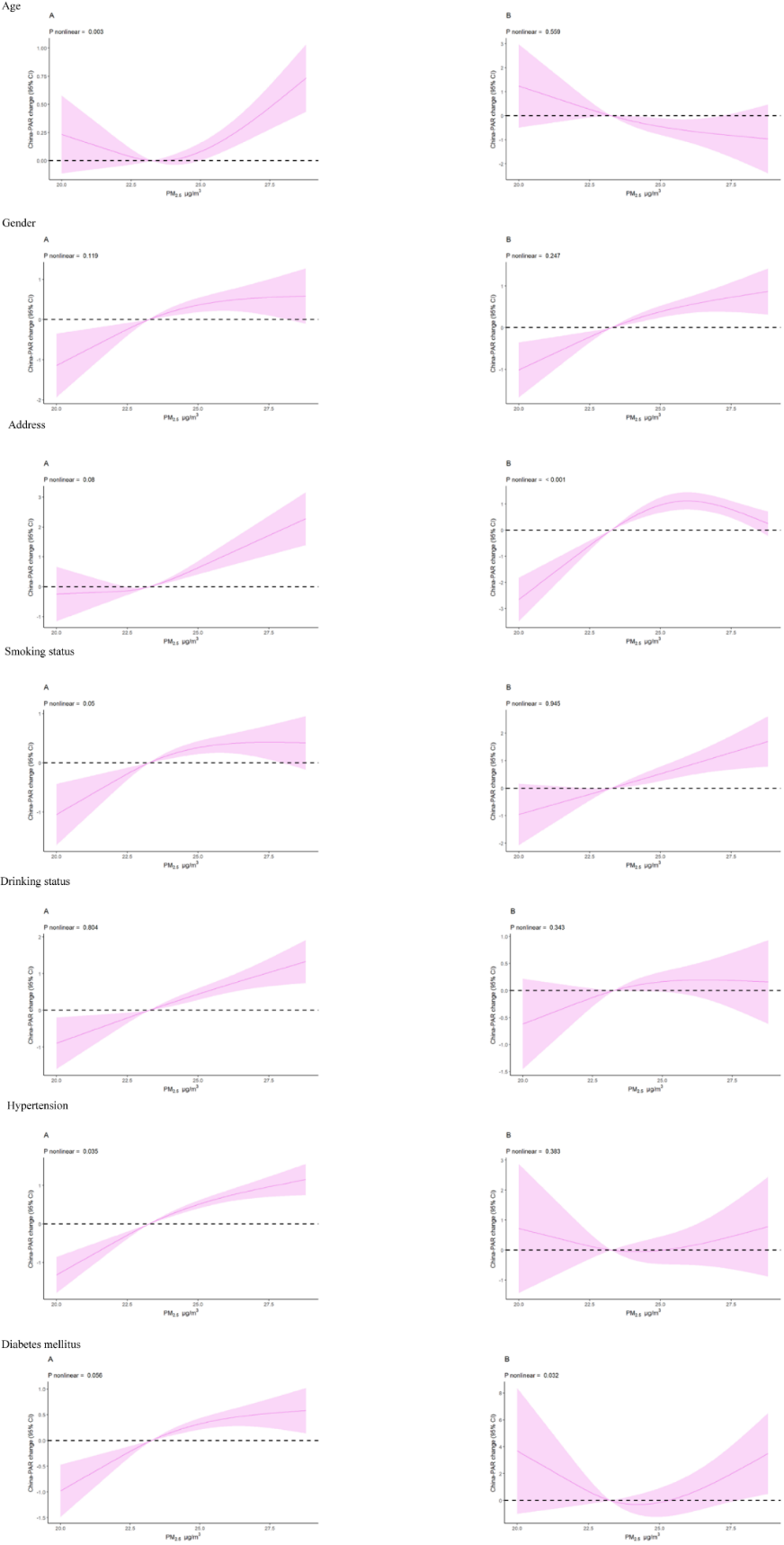


**Fig.S1. The restricted cubic spline for the association between PM_2.5_ exposure levels and China-PAR scores in stratified analyses.** Adjusted restricted cubic spline was used to estimate the exposure-response relationship (solid line) and its 95% confidence interval (shaded). All models were adjusted for marital status, education level, drinking status, and per capita disposable income. Abbreviations: PM_2.5_, fine particulate matter; China-PAR, risk prediction of atherosclerotic cardiovascular disease in China; CI, confidence interval.
